# Eight‐year follow‐up outcome of subthalamic deep brain stimulation for Parkinson’s disease: Maintenance of therapeutic efficacy with a relatively low levodopa dosage and stimulation intensity

**DOI:** 10.1111/cns.13713

**Published:** 2021-08-05

**Authors:** Lulu Jiang, Wanru Chen, Qiyu Guo, Chao Yang, Jing Gu, Wenbiao Xian, Yanmei Liu, Yifan Zheng, Jing Ye, Shaohua Xu, Yu Hu, Lei Wu, Jie Chen, Hao Qian, Xiaoli Fu, Jinlong Liu, Ling Chen

**Affiliations:** ^1^ Department of Neurology Guangdong Provincial Key Laboratory of Diagnosis and Treatment of Major Neurological Diseases National Key Clinical Department and Key Discipline of Neurology The First Affiliated Hospital, Sun Yat‐sen University Guangzhou China; ^2^ Department of Neurology Sun Yat‐sen Memorial Hospital, Sun Yat‐sen University Guangzhou China; ^3^ Department of Neurosurgery The First Affiliated Hospital, Sun Yat‐sen University Guangzhou China; ^4^ Department of Medical Statistics and Epidemiology School of Public Health Sun Yat‐sen University Guangzhou China

**Keywords:** deep brain stimulation, follow‐up Studies, Parkinson's disease, subthalamic nucleus

## Abstract

**Aims:**

This follow‐up study aimed to examine the 8‐year efficacy and safety of subthalamic nucleus (STN) deep brain stimulation (DBS) for patients with Parkinson's disease (PD) in southern China.

**Methods:**

The follow‐up data of 10 patients with PD undergoing STN‐DBS were analyzed. Motor symptoms were assessed before and 1, 3, 5, and 8 years after the surgery with stimulation‐on in both off‐medication (off‐med) and on‐medication (on‐med) status using the Unified Parkinson's disease Rating Scale Part III. The quality of life was assessed using the 39‐item Parkinson's Disease Questionnaire. The sleep, cognition, and emotion were evaluated using a series of nonmotor scales. Levodopa equivalent daily dose (LEDD) and stimulation parameters were recorded at each follow‐up.

**Results:**

The motor symptoms were improved by 50.9%, 37.7%, 36.7%, and 37.3% in 1, 3, 5, and 8 years, respectively, in the off‐med / stimulation‐on status compared with the baseline. The quality of life improved by 39.7% and 56.1% in 1 and 3 years, respectively, but declined to the preoperative level thereafter. The sleep, cognition, and emotion were mostly unchanged. LEDD reduced from 708.1 ± 172.5 mg to 330 ± 207.8 mg in 8 years. The stimulation parameters, including amplitude, pulse width, and frequency, were 2.77 ± 0.49 V, 71.3 ± 12.8 μs, and 121.5 ± 21 Hz, respectively, in 8 years.

**Conclusion:**

Long‐term therapeutic efficacy of STN‐DBS could be achieved even with relatively low stimulation intensity and medication dosage for PD patients in southern China. Motor improvement and medication reduction were maintained through the 8‐year follow‐up, but improvement in quality of life lasted for only 3 years. No definite changes was found in nonmotor symptoms after STN‐DBS.

## Graphical abstract

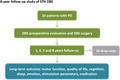

## 1 INTRODUCTION

Parkinson's disease (PD) is the most common neurodegenerative disease after Alzheimer's disease. As a surgical treatment, deep brain stimulation (DBS) has gained widespread popularity since it was introduced in the 1970s and become the treatment of choice for advanced PD. Recent years have seen breakthroughs in electrode and battery designs, new stimulation paradigms, adaptive closed‐loop stimulation, and sensing technologies, which hold a promise for higher efficacy and tolerability of DBS.^1^ The efficacy of DBS for PD is well‐established for up to 1 or 2 years, but long‐term outcome data are still limited.^2^ Both short‐term and long‐term efficacies of DBS have been reported in Western countries.^3^, ^4^, ^5^, ^6^ However, the long‐term efficacy of DBS has not been thoroughly studied in China, although China has the largest proportion of patients with PD worldwide. Furthermore, with the influence of racial, cultural, and probably genetic differences, patients with PD in China are characterized by lower body weight, lower dosages of levodopa, and lower prevalence of dyskinesia. Whether these factors have an impact on the management, efficacy, and safety profiles of DBS in Chinese patients, especially in the long‐term, is a practical but unclear problem for Chinese neurologists. This study aimed to report the findings of 1‐, 3‐, 5‐, and 8‐year follow‐ups for 10 consecutive patients with PD, who underwent bilateral subthalamic nucleus (STN) DBS in the First Affiliated Hospital, Sun Yat‐sen University.

## 2 MATERIALS AND METHODS

A total of 20 patients diagnosed with PD according to the criteria of the United Kingdom Parkinson's Disease Society Brain Bank underwent bilateral STN‐DBS in the center during 2008–2010. The inclusion and exclusion criteria for DBS surgery were as stated in a previous study.^7^ A month before undergoing surgery, the anti‐parkinsonian drugs remained unchanged to keep the whole condition stable. The patients were evaluated preoperatively (baseline assessment) and in 1, 3, 5, and 8 years postoperatively (follow‐up assessment). The “on” and “off” motor states were recorded on video at the baseline and each follow‐up.

### 2.1 Motor and nonmotor assessment

Preoperative motor function was evaluated with the Unified Parkinson's disease Rating Scale Part III (UPDRS III) in a levodopa challenge test. Details of the test were reported in our previous study.^7^ Follow‐up evaluations were performed in the off‐med and on‐med states with the stimulator on in 1, 3, 5, and 8 years. The following nonmotor symptoms were also assessed: quality of life by the 39‐item Parkinson's Disease Questionnaire (PDQ‐39), sleep by the Parkinson's Disease Sleep Scale Chinese Version (PDSS‐CV), Pittsburgh Sleep Quality Index (PSQI), and Epworth Sleepiness Scale (ESS), cognition by the Mini‐Mental State Examination (MMSE) and the Montreal Cognitive Assessment (MoCA), and emotion by the Hamilton Anxiety Scale (HAMA) and the Hamilton Depression Scale (HAMD).

### 2.2 Surgical procedure

Before the surgery, each patient underwent a nonstereotactic brain magnetic resonance imaging scan and a stereotactic brain computed tomography (CT) scan, which were then fused together to facilitate trajectory planning. As previously reported, implantation of electrodes (Model 3389; Medtronic, MN, USA) into STN was carried out under local anesthesia, with the ideal target determined by the microelectrode recording and intraoperative stimulation. The implantable pulse generator (IPG) (Kinetra, Medtronic) was implanted under general anesthesia. The final position of the electrodes was confirmed with a brain CT scan the next day. When the battery of the IPG wore off 4–5 years after the surgery, a new rechargeable (Activa RC, Medtronic) or nonrechargeable IPG (Activa PC, Medtronic) was replaced.

### 2.3 Programming

The IPG was switched on 1 month after the surgery, allowing for the microlesion effect to fade away. The procedure for initial programming was detailed in the previous report.^7^ The initial stimulation parameters, including pulse width, frequency, and voltage, were set at 60 μs, 130 Hz, and 0.8–1.5 V, respectively, in a single monopolar configuration with the IPG as an anode and the optimal contact as a cathode. The patients were asked to return in 2 weeks for parameter adjustment. Thereafter, they made a programming appointment as needed. The stimulation parameters and medications were adjusted by experienced neurologists. Before each follow‐up, the medications and parameters remained unchanged for at least 1 month. Levodopa equivalent daily dose (LEDD), stimulation parameters, and adverse events (AEs) were recorded at each follow‐up.

### 2.4 Statistical analyses

Continuous variables were presented as mean ± standard deviation. The Shapiro‐Wilk test for normality were used to assess data distribution. The baseline characteristics were compared between patients who completed the total follow‐up (*n *= 10) and those lost during the 8‐year follow‐up (*n* = 10) using the independent‐samples *t* test and Mann–Whitney *U* test. The Fisher exact test was adopted for categorical variables. The weight and body mass index (BMI) at the baseline and in 8 years for the tracked group were compared using the paired‐samples *t* test. The overtime differences in UPDRS scores, nonmotor measurements, and LEDD were analyzed using the analysis of variance (ANOVA) for repeated measures (only data of patients who completed the 8‐year follow‐up were analyzed). The sphericity assumption was evaluated using the Mauchly's test and adjusted using the Greenhouse‐Geisser correction. The least significant difference (LSD) *t* test was used for the post hoc multiple comparison of data at the baseline and in 1, 3, 5, and 8 years. A two‐sided *p* < 0.05 was considered as statistically significant. Statistical analyses were performed using SPSS software version 22.0 (SPSS Inc.).

## 3 RESULTS

### 3.1 Study population

A total of 20 patients with PD (11 male and 9 female) who underwent bilateral STN‐DBS were included in this study. Their mean age at surgery was 59.3 ± 8.7 years, and the disease duration was 9.6 ± 2.8 years. All of them achieved more than 30% improvement in motor symptoms in the preoperative levodopa challenge test. At the end, only 10 patients completed the 8‐year follow‐up. The others were lost: five died of diseases not related to DBS; one immigrated overseas; one ran out of battery but could not afford a new one; one lived in a province too far away; one developed dementia and stayed in the nursing home; and one lived alone and was unable to return without help. Nine of the drop‐out cases finished the baseline, 1‐year and 3‐year follow‐ups. Only 2 finished the 5‐year follow‐up, and none finished the 8‐year follow‐up. Except for age, no significant difference was found in baseline characteristics between the tracked and lost patients (Table 1). The average weight of the tracked group at the baseline and in 8 years was 63.0 ± 9.8 kg and 63.9 ± 12.7 kg (*p *= 0.610), and the average BMI was 22.6 ± 2.7 and 22.9 ± 3.7 (*p* = 0.623), respectively.

**TABLE 1:** Baseline characteristics of the 20 patients with PD

Characteristic	Tracked patients (*N* = 10)	Lost patients (*N* = 10)	*p*
Age (year)^a^	55.4 ± 9.9	63.1 ± 5.2	0.048
Male (%)^b^	70	40	0.370
History of the previous pallidotomy^b^	1	2	1.000
Duration of PD ^a^	8.9 ± 2.1	10.2 ± 3.3	0.312
“Off” H–Y state^c^	2.9 ± 0.2	3.1 ± 0.6	0.436
“On” H–Y state^c^	2.2 ± 0.5	2.3 ± 0.6	0.739
Improvement in challenge test (%)^a^	66.3 ± 12.1	66.1 ± 13.3	0.979
LEDD (mg)	710.6 ± 176.9	778.5 ± 245.7	0.487

Note

Values are presented as mean ± standard deviation.

Abbreviations: H‐Y, Hoehn and Yahr stages; LEDD, levodopa equivalent daily dose; PD, Parkinson's disease.

^a^ These variables were analyzed using the independent‐samples *t* test.

^b^ These variables were analyzed using the Fisher exact test (two‐sided).

^c^ These variables were analyzed using the Mann–Whitney *U* test.

### 3.2 Motor outcome

Compared with the preoperative off‐med state, motor symptoms (evaluated with UPDRS III) significantly improved from the baseline to 1, 3, 5, and 8 years postoperatively by 50.9%, 37.7%, 36.7%, and 37.3%, respectively (Table 2). Tremor and rigidity showed a prominent and sustained improvement of more than 50% during the 8‐year follow‐up. However, the improvement in bradykinesia and axial symptoms lasted only for 5 and 3 years, respectively. As for the on‐med state, the total motor scores remained stable at the baseline and in 1, 3, and 5 years, but deteriorated by 46.8% in 8 years, due to the worsening of bradykinesia (deteriorated by 103.3%) and axial symptoms (deteriorated by 113.2%). In contrast, the rigidity showed a remarkable improvement of 71.2%, 68.3%, and 49.0% in 3, 5, and 8 years, respectively. Scores of UPDRS I (mentation, behavior, and mood) and UPDRS II (activities of daily living) worsened starting from 5 years postoperatively.

**TABLE 2:** UPDRS scores at the baseline and in 1, 3, 5, and 8 years postoperatively in off‐med / stimulation‐on and on‐med / stimulation‐on

UPDRS subscale	Item number	Range of score	State	Baseline (*n* = 10)	1 year (*n* = 10)	3 years (*n* = 10)	5 years (*n* = 10)	8 years (*n* = 10)
UPDRS I	1–4	0–16	/	1.7 ± 1.2	1.9 ± 2.8	2.4 ± 2.3	2.7 ± 2.5	3.2 ± 2.9
UPDRS II	5–17	0–52	Off	14.8 ± 5.3	11.4 ± 5.5*	14.0 ± 5.9	17.3 ± 7.3	18.7 ± 7.1
			On	4.7 ± 4.3	5.5 ± 4.6^**^	5.3 ± 5.2*	8.7 ± 4.4^**^	13.2 ± 7.5^§§^
UPDRS III								
Total score	18–31	0–108	Off	46.8 ± 6.8	23.0 ± 7.8^§§§*^	29.2 ± 9.1^§§^	29.6 ± 8.0^§§^	29.3 ± 8.7^§§^
			On	15.4 ± 6.4	14.2 ± 5.7^**^	14.3 ± 8.0*	17.8 ± 6.1*	22.6 ± 7.1^§^
Tremor	20–21	0–28	Off	6.6 ± 4.9	3.3 ± 3.3^§^	4.4 ± 2.8^**^	2.6 ± 2.1^§^	2.1 ± 2.8^§^
			On	0.4 ± 0.7	1.5 ± 2.0	1.0 ± 1.3	0.7 ± 1.3	0.6 ± 1.0
Rigidity	22	0–20	Off	11.7 ± 2.4	5.4 ± 2.9^§§§^	4.3 ± 3.6^§§§^	4.8 ± 4.0^§§§^	4.4 ± 2.2^§§§^
			On	5.2 ± 2.1	4.1 ± 2.4	1.5 ± 1.2^§§§*^	1.7 ± 1.4^§§§^	2.7 ± 1.9^§§^
Bradykinesia	23–26, 31	0–36	Off	18.9 ± 4.2	9.2 ± 2.9^§§§**^	12.8 ± 4.2^§^	13.7 ± 4.5^§^	14.6 ± 5.3
			On	6.1 ± 3.1	4.9 ± 3.2^**^	7.0 ± 5.1*	9.1 ± 4.1*	12.4 ± 4.8^§^
Axial symptoms	27–30	0–16	Off	6.2 ± 1.7	3.0 ± 2.1^§§*^	4.4 ± 1.3^§^	5.0 ± 2.4	4.9 ± 2.3
			On	1.9 ± 1.9	2.3 ± 1.6^**^	2.3 ± 1.2*	3.3 ± 2.2	4.05 ± 2.0^§^

Note

Values are presented as mean ± standard deviation. The ANOVA for repeated measures showed significant time effects for OFF‐UPDRS II (*F* = 4.143, *p* = 0.024), ON‐UPDRS II (*F* = 7.558, *p* = 0.003), OFF‐UPDRS III (*F* = 17.538, *p* < 0.001) and ON‐UPDRS III (*F* = 4.023, *p* = 0.008) scores, indicating changes during the follow‐up period. Post‐hoc multiple comparison was performed using the LSD *t* test.

Abbreviations: UPDRS, Unified Parkinson's Disease Rating Scale, with higher scores indicating worse functioning.

§ *p* < 0.05 compared to baseline, ^§§^ *p* < 0.01 compared to baseline, ^§§§^ *p* < 0.001 compared to baseline.

* *p* < 0.05 compared to 8 years, ^**^ *p* < 0.01 compared to 8 years, ^***^ *p* < 0.001 compared to 8 years.

### 3.3 Nonmotor outcome

The mean score of the PDQ‐39 summary index (PDQ‐39 SI) at the baseline was 33.7 ± 12.7. The percentage change in the quality of life from the baseline to 1, 3, 5, and 8 years was 39.7% (*p* = 0.009), 56.1% (*p* < 0.001), 18.8% (*p* = 0.061), and −4.1% (*p* = 0.769), respectively, indicating an improvement in the first 3 years, followed by a drop to the baseline thereafter. The dimension scores of mobility, ADL, emotion, and stigma significantly improved from the baseline to 3 years by 57.8%, 61.2%, 72.6%, and 73.7%, respectively. Significant deterioration was observed in the dimension scores of cognition (−56.7%) in 8 years. The scores of other dimensions were mostly stable over time. The HAMA scores remarkably improved in 1 year compared with the baseline but gradually went back to the baseline afterward. A significant improvement in MoCA scores was seen in 3 years, but disappeared starting from 5 years postoperatively. No other significant differences were observed over time for sleep (PDSS‐CV, PSQI, and ESS scores), cognition (MMSE scores), and emotion (HAMA scores) (Table 3).

**TABLE 3:** Nonmotor scores at the baseline and in 1, 3, 5, and 8 years postoperatively

Items	Range of score	Baseline	1 year	3 years	5 years	8 years
Quality of life						
PDQ−39 SI	0–100	33.7 ± 12.7	20.3 ± 10.6^§§**^	14.8 ± 12.1^§§§***^	27.4 ± 10.8*	35.1 ± 13.7
Mobility	0–100	43.3 ± 20.5	26.0 ± 15.1^§§**^	18.3 ± 15.1^§§**^	30.5 ± 22.8^**^	52.8 ± 33.2
ADL	0–100	42.9 ± 17.8	22.9 ± 17.8^§^	16.7 ± 20.3^§§*^	22.1 ± 15.5^§§^	32.5 ± 21.8
Emotion	0–100	39.6 ± 21.4	13.8 ± 11.6^§§**^	10.8 ± 13.8^§§*^	24.6 ± 15.5	30.0 ± 18.3
Stigma	0–100	35.6 ± 25.2	20.6 ± 18.2	9.4 ± 16.5^§§^	17.5 ± 16.6^§^	18.1 ± 30.4
Social support	0–100	17.5 ± 31.0	4.2 ± 8.1*	0.8 ± 2.6^***^	7.5 ± 12.1	14.2 ± 16.2
Cognition	0–100	31.9 ± 14.9	29.4 ± 20.2^**^	22.5 ± 24.0^***^	38.8 ± 25.8	50.0 ± 29.2^§^
Communication	0–100	28.3 ± 19.7	17.5 ± 12.7^**^	15.0 ± 17.5^***^	42.5 ± 20.6	47.5 ± 21.5
Bodily discomfort	0–100	30.8 ± 25.5	28.3 ± 17.2*	25.0 ± 21.5*	35.8 ± 18.4	35.8 ± 15.2
Sleep						
PDSS‐CV	0–150	102.9 ± 20.1	114.8 ± 16.9	109.1 ± 28.3	108.7 ± 22.3	97.9 ± 23.8
PSQI	0–42	11.0 ± 4.3	8.1 ± 3.9	8.2 ± 5.9	10.3 ± 5.5	10.8 ± 4.7
ESS	0–24	9.2 ± 3.4	9.2 ± 6.7	7.7 ± 7.7	9.0 ± 7.5	13.1 ± 6.9
Cognition						
MMSE	0–30	28.9 ± 0.8	28.9 ± 0.8	29.2 ± 0.8	28.7 ± 1.1	28.1 ± 1.9
MoCA	0–30	24.1 ± 3.2	24.7 ± 1.4	27.1 ± 2.0^§*^	24.9 ± 3.4	23.9 ± 3.0
Emotion						
HAMD	0–76	9.7 ± 6.5	5.7 ± 3.8	8.7 ± 7.8	7.9 ± 7.9	10.8 ± 8.5
HAMA	0–56	8.8 ± 4.9	5.2 ± 3.2^§§*^	5.4 ± 6.2	6.3 ± 6.8	9.5 ± 6.9

Note

Values are presented as mean ± standard deviation. The ANOVA for repeated measures showed significant time effects for PDQ‐39 SI (*F* = 17.366, *p* < 0.001), MoCA (*F* = 3.756, *p* = 0.024) and HAMA (*F* = 2.973, *p* = 0.032), indicating changes during the follow‐up period. Post‐hoc multiple comparison was performed using the LSD *t* test.

Abbreviations: ADL, activities of daily living; ESS, Epworth Sleepiness Scale; HAMA, Hamilton Anxiety Scale; HAMD, Hamilton Depression Scale; MMSE, Mini‐Mental State Examination; MoCA, Montreal Cognitive Assessment; PDQ‐39 SI, 39‐item Parkinson's Disease Questionnaire summary index; PDSS‐CV, Parkinson's Disease Sleep Scale Chinese Version; PSQI, Pittsburgh Sleep Quality Index. Higher scores indicate better functioning in PDSS‐CV, MMSE, and MoCA, but worse functioning in PDQ‐39 SI, PSQI, ESS, HAMD, and HAMA.

§ *p* < 0.05 compared to baseline, ^§§^ *p* < 0.01 compared to baseline, ^§§§^ *p* < 0.001 compared to baseline.

* *p* < 0.05 compared to 8 years, ^**^ *p* < 0.01 compared to 8 years, ^***^ *p* < 0.001 compared to 8 years.

### 3.4 Medication

The mean LEDD decreased from 708.1 ± 172.5 mg at the baseline to 382.5 ± 188.6 mg in 1 year (46.0%, *p* = 0.001), 322.5 ± 168.5 mg in 3 years (54.5%, *p* < 0.001), 350.6 ± 162.9 mg in 5 years (50.5%, *p* < 0.001), and 330 ± 207.8 mg in 8 years (53.4%, *p* < 0.001) postoperatively. No statistically significant differences were found between postoperative LEDDs at different time points (*p* > 0.05). In 8 years, levodopa, dopamine agonists, selegiline, and amantadine was taken by eight, six, one, and two patients, respectively. One patient withdrew all medications starting from 1 year postoperatively, and one took only levodopa.

### 3.5 Programming

Multiple stimulation settings were observed in 8 years, including single monopolar, double monopolar, bipolar, and interleaving configurations. The amplitude gradually increased from 1 to 8 years, while the frequency was stable in 1, 3, and 5 years but decreased in 8 years; the pulse width remained unchanged (Table 4). Of the 10 successfully tracked patients, 8 had their IPG replaced once, and 2 had it twice. The average battery life was 5.9 ± 1.2 years during their first replacement, with a battery voltage of 2.45 ± 0.23 V. Those who could not be tracked never came back for IPG replacement.

**TABLE 4:** Stimulation parameters in 1, 3, 5, and 8 years postoperatively

Parameters	1 year^a^ (*n* = 10)	3 years^b^ (*n* = 10)	5 years^c^ (*n* = 10)	8 years^d^ (*n* = 10)
Amplitude (volt)	2.23 ± 0.31^c, d^	2.41 ± 0.44^c, d^	2.63 ± 0.37^a, b^	2.77 ± 0.49^a, b^
Pulse width (μs)	72.0 ± 15.8	73.5 ± 18.1	75.0 ± 18.2	71.3 ± 12.8
Frequency (Hz)	147.5 ± 15.8^d^	145.0 ± 23.1^d^	142.5 ± 19.6^d^	121.5 ± 21.3^a, b, c^
Stimulation patterns (monopolar/bipolar/interleaving)	9/1/0	9/1/0	9/1/0	8/0/2

Note

Values are presented as mean ±standard deviation (SD). The ANOVA for repeated measures showed significant time effects for amplitude (*F* = 12.193, *p* < 0.001) and frequency (*F* = 13.286, *p* < 0.001), indicating changes during the follow‐up period. Post‐hoc multiple comparison was performed using the LSD *t* test. ^a, b, c, d^The presence of a statistically significant difference among the conditions (*p* < 0.05).

### 3.6 AEs and safety

AEs observed during the 8‐year follow‐up were recorded for all patients. Troublesome dyskinesia, gait deterioration and falls, dysarthria, and drooling were the second most frequent AEs, following the microlesion effect, which was found in every patient after the surgery. No serious AEs related to the surgery or the DBS device were noted. However, five deaths were caused by diseases unrelated to DBS, including two cases of pulmonary infection, one case of rectal cancer, one case of stroke, and one case of unknown cause (the patient died while sleeping).

## 4 DISCUSSION

In China, only a few studies have presented the long‐term outcome of STN‐DBS in PD patients. Li et al. reported an improvement of over 50% in off‐medication motor scores (UPDRS III) and activities of daily living scores (UPDRS II) at 5 years.^8^ As was shown by other domestic studies, the motor benefit of STN‐DBS still persisted after 5 years.^9^, ^10^, ^11^ For patients with early‐onset PD, axial symptoms were responsive to STN‐DBS even at 13 years.^12^ However, all these studies mainly focused on motor function. Nonmotor outcome were limited, although some short‐term results has been reported.^13^ To help fill the gap, we reported our 8‐year follow‐up data of STN‐DBS after a thorough assessment of both motor and nonmotor symptoms.

The drop‐out rate of our study was high in 8 years. The older patients tended to drop out more easily, probably because they had a higher risk of having other diseases that might worsen their mobility or lead to death. Postoperative weight gain has been reported in many studies.^14^, ^15^, ^16^ However, no difference was found between weight at the baseline and that in 8 years, suggesting a reduction in weight as the disease progressed, offsetting the weight gain in the first few years.

According to the off‐med motor scores, STN‐DBS alone significantly improved motor symptoms up to 8 years postoperatively in this study. Tremor and rigidity showed the best response to STN‐DBS while bradykinesia and axial symptoms showed a diminishing response, which was also found by other teams.^17^, ^18^ In the on‐med state, the motor scores started to worsen after 5 years. One possible cause of the deterioration was decreased levodopa responsiveness.^19^ With a longer follow‐up, the disease progressed and levodopa‐resistant symptoms, such as axial symptoms, developed. Axial symptoms are related to not only the dopaminergic pathway but also other transmitter pathways including noradrenergic, acetylcholinergic, and serotoninergic pathways.^20^ In the present study, another possible cause of on‐med deterioration might be an increased but unmet need for dopaminergic medication. Both the patients and the physicians in our center preferred programming to medication adjustment when parkinsonian symptoms worsened. Hence, LEDD remained at a relatively low level even in 8 years. The complications related to medications might be reduced, but the risk of undertreatment of levodopa‐responsive symptoms could increase. Keeping the LEDD at a lower level is not always a good choice. An increase in LEDD should be considered when symptom control is not satisfactory.

The overall quality of life (PDQ‐39 SI scores) improved in the first 3 years postoperatively. The mobility, ADL, emotion, and stigma showed a remarkable improvement up to 5 years, which might probably benefit from improved motor symptoms. However, this improvement was lost during the 8‐year follow‐up, which might have resulted from the deterioration of on‐med motor function and lower battery. The dimension score of cognition, comprising four items including somnolence, concentration, memory, and distressing dreams/hallucinations, also worsened in 8 years, with the first and the fourth items as the main contributors. Other nonmotor symptoms, including sleep, cognition, and emotion, remained mostly stable over time. Given the selection bias, deterioration of these nonmotor aspects, especially cognition, might be found in patients lost to follow‐up, with age being a risk factor of dementia. Studies about changes in nonmotor symptoms after STN‐DBS have provided conflicting results including improvements, a lack of changes, and worsening (for review, see Ref. 21 and 22). Improvement of nonmotor symptoms, for example, sleep, might be either directly or indirectly due to motor benefit or reduction of dopaminergic drug load.^21^ Decline in postoperative executive function, especially verbal fluency, has been consistently reported. This adverse change might be due to insertion and location of the electrodes.^22^ It has been shown that medially located electrodes on the left STN were associated with a significantly higher risk of speech deterioration than electrodes within the nucleus.^23^ However, in most cases, this decline did not have a significant impact on the global cognitive function and the patients’ quality of life.^24^ In addition, dementia prevalence and incidence after STN‐DBS were not higher than those reported in the general PD population.^25^ Postoperative psychiatric complications, for example, depression, apathy, hypomania, impulse control disorders, and psychosis, have been documented. The use of or changes in dopaminergic medications, the effect of stimulation on the limbic‐associative territory of STN, and a history of these psychiatric conditions might be possible contributors.^22^ Compared with the baseline, postoperative LEDD decreased remarkably by 46.0% in 1 year, 54.5% in 3 years, 50.5% in 5 years, and 53.4% in 8 years. Although the motor improvement and percentage reduction of LEDD in 8 years were similar to those reported by other centers,^18^, ^26^, ^27^ the mean preoperative LEDD in the present study was lower (708.1 mg vs 890.4–1471.1 mg). Since the Chinese treatment guidelines for PD recommend the smallest possible dose for satisfactory symptom control to avoid motor complications, anti‐parkinsonian drugs in China are taken at doses lower than those recommended for Western patients.^28^ Furthermore, there might be a difference in the actual need for levodopa among different patient populations. According to DBS follow‐up studies from various regions, the preoperative LEDD for Asian patients with advanced PD ranged from 670 to 1066 mg,^29^, ^30^, ^31^, ^32^ in contrast to 890 to 1471 mg for Western patients.^3^, ^4^, ^18^, ^27^, ^33^ A pharmacokinetic study demonstrated an inverse correlation between body weight and peripheral pharmacokinetic parameters of levodopa, namely the plasmatic levodopa area under the curve and elimination half‐life, suggesting higher plasma levels in lower‐weight patients with PD taking the same dose.^34^ Hence, the low body weight of our cohort (65.4 ± 7.8 kg for men and 50.7 ± 7.0 kg for women) probably contributed to the lower LEDD.

Similarly, the average stimulation parameters (2.77 V, 71.3 μs, and 121.5 Hz) in the present study also seemed to be lower than those reported in Western countries (3.1–3.5 V, 62–90 μs, and 142–161 Hz),^5^, ^18^, ^35^, ^36^ which might be due to the difference in weight, considering the same anti‐parkinsonian effect of electrical stimulation and levodopa. However, the value of the total electrical energy delivered was needed for the precise comparison of the stimulation intensity.^37^ A slight decrease in frequency was seen in 8 years, along with the worsening of axial symptoms. High‐frequency stimulation could aggravate the axial symptoms, while low‐frequency (60 Hz) stimulation might have an opposite effect.^38^, ^39^, ^40^ However, the benefit of low frequency was temporary and at the cost of worsening other motor symptoms. Therefore, a compromising approach was adopted and a frequency between 90 and 125 Hz was applied. It turned out that the patients were slightly more comfortable using this relatively low frequency, which was consistent with the findings of another follow‐up study in China.^12^ Five patients used the common monopolar stimulation, three needed the more intense double monopolar setting for symptom control, and two switched to the interleaving stimulation because of dyskinesia and eyelid opening apraxia 8 years after the surgery.

Long‐term stimulation was well‐tolerated in this study. No AE related to operation or device was observed. All five deaths were caused by conditions unrelated to the surgery or stimulation. Dyskinesia, gait deterioration and falls, dysarthria, and drooling were the most frequent AEs. In most cases, dyskinesia could be handled by reducing levodopa, adjusting stimulation parameters, or adding amantadine. Gait deterioration, dysarthria, and drooling are the features of advanced PD,^41^ and can also be caused or worsened by high‐frequency stimulation, as mentioned previously. In addition to adjusting medication and stimulation parameters, rehabilitation treatment may also be helpful in managing axial symptoms.^42^

The limitations of this study included the small sample size, unblinded and monocenter study design, high drop‐out rate, and lack of stimulation‐off motor assessment due to the patients' intolerance of this condition.

## 5 CONCLUSIONS

This study showed that the therapeutic efficacy of STN‐DBS could be maintained for at least 8 years for patients with PD in southern China, with a relatively low medication dosage and stimulation intensity. However, the initial benefit of DBS decreased as the disease progressed. The improvement in the quality of life was lost after 3 years, and the on‐med motor function worsened in 8 years. The changes in nonmotor symptoms were not significant. The programming of late‐staged patients with dominant axial symptoms is tricky, although a small decrease in frequency may be helpful. Medication adjustment and physical therapy should be considered when programming yields limited or no improvement.

## CONFLICT OF INTEREST

The authors report no conflict of interest concerning the materials or methods used in this study or the findings specified in this paper.

## ETHICS APPROVAL AND CONSENT TO PARTICIPATE

The study was approved by the Medical Ethical Committee of the First Affiliated Hospital, Sun Yat‐sen University. Signed informed consent was obtained from all patients before their participation in the study.

## DATA AVAILABILITY STATEMENT

The data that support the findings of this study are available from the corresponding author upon reasonable request.
